# Association between circulating inflammatory markers and adult cancer risk: a Mendelian randomization analysis

**DOI:** 10.1016/j.ebiom.2024.104991

**Published:** 2024-02-01

**Authors:** James Yarmolinsky, Jamie W. Robinson, Daniela Mariosa, Ville Karhunen, Jian Huang, Niki Dimou, Neil Murphy, Kimberley Burrows, Emmanouil Bouras, Karl Smith-Byrne, Sarah J. Lewis, Tessel E. Galesloot, Lambertus A. Kiemeney, Sita Vermeulen, Paul Martin, Demetrius Albanes, Lifang Hou, Polly A. Newcomb, Emily White, Alicja Wolk, Anna H. Wu, Loïc Le Marchand, Amanda I. Phipps, Daniel D. Buchanan, The International Lung Cancer Consortium, Maria Teresa Landi, Victoria Stevens, Ying Wang, Demetrios Albanes, Neil Caporaso, Paul Brennan, Christopher I. Amos, Sanjay Shete, Rayjean J. Hung, Heike Bickeböller, Angela Risch, Richard Houlston, Stephen Lam, Adonina Tardon, Chu Chen, Stig E. Bojesen, Mattias Johansson, H-Erich Wichmann, David Christiani, Gadi Rennert, Susanne Arnold, John K. Field, Loic Le Marchand, Olle Melander, Hans Brunnström, Geoffrey Liu, Angeline Andrew, Lambertus A. Kiemeney, Hongbing Shen, Shan Zienolddiny, Kjell Grankvist, Mikael Johansson, M. Dawn Teare, Yun-Chul Hong, Jian-Min Yuan, Philip Lazarus, Matthew B. Schabath, Melinda C. Aldrich, The PRACTICAL Consortium, Rosalind A. Eeles, Christopher A. Haiman, Zsofia Kote-Jarai, Fredrick R. Schumacher, Sara Benlloch, Ali Amin Al Olama, Kenneth R. Muir, Sonja I. Berndt, David V. Conti, Fredrik Wiklund, Stephen Chanock, Ying Wang, Catherine M. Tangen, Jyotsna Batra, Judith A. Clements, Henrik Grönberg, Nora Pashayan, Johanna Schleutker, Demetrius Albanes, Stephanie J. Weinstein, Alicja Wolk, Catharine M.L. West, Lorelei A. Mucci, Géraldine Cancel-Tassin, Stella Koutros, Karina Dalsgaard Sørensen, Eli Marie Grindedal, David E. Neal, Freddie C. Hamdy, Jenny L. Donovan, Ruth C. Travis, Robert J. Hamilton, Sue Ann Ingles, Barry S. Rosenstein, Yong-Jie Lu, Graham G. Giles, Robert J. MacInnis, Adam S. Kibel, Ana Vega, Manolis Kogevinas, Kathryn L. Penney, Jong Y. Park, Janet L. Stanfrod, Cezary Cybulski, Børge G. Nordestgaard, Sune F. Nielsen, Hermann Brenner, Christiane Maier, Christopher J. Logothetis, Esther M. John, Manuel R. Teixeira, Susan L. Neuhausen, Kim De Ruyck, Azad Razack, Lisa F. Newcomb, Davor Lessel, Radka Kaneva, Nawaid Usmani, Frank Claessens, Paul A. Townsend, Jose Esteban Castelao, Monique J. Roobol, Florence Menegaux, Kay-Tee Khaw, Lisa Cannon-Albright, Hardev Pandha, Stephen N. Thibodeau, David J. Hunter, Peter Kraft, William J. Blot, Elio Riboli, Sizheng Steven Zhao, Dipender Gill, Stephen J. Chanock, Mark P. Purdue, George Davey Smith, Paul Brennan, Karl-Heinz Herzig, Marjo-Riitta Järvelin, Chris I. Amos, Rayjean J. Hung, Abbas Dehghan, Mattias Johansson, Marc J. Gunter, Kostas K. Tsilidis, Richard M. Martin

**Affiliations:** a MRC Integrative Epidemiology Unit, University of Bristol, Bristol, UK; b Population Health Sciences, Bristol Medical School, University of Bristol, Bristol, UK; c Department of Epidemiology and Biostatistics, School of Public Health, Imperial College London, St Mary's Campus, London, UK; d Genomic Epidemiology Branch, International Agency for Research on Cancer (IARC/WHO), Lyon, France; e Research Unit of Population Health, Faculty of Medicine, University of Oulu, Oulu, Finland; f Research Unit of Mathematical Sciences, University of Oulu, Oulu, Finland; g Singapore Institute for Clinical Sciences (SICS), Agency for Science, Technology and Research (A∗STAR), Singapore, Singapore; h Nutrition and Metabolism Branch, International Agency for Research on Cancer (IARC/WHO), Lyon, France; i Department of Hygiene and Epidemiology, University of Ioannina School of Medicine, Ioannina, Greece; j The Cancer Epidemiology Unit, Nuffield Department of Population Health, University of Oxford, Oxford, UK; k Radboud University Medical Center, Nijmegen, The Netherlands; l Department for Health Evidence, Radboud University Medical Center, Nijmegen, The Netherlands; m School of Biochemistry, Biomedical Sciences Building, University of Bristol, University Walk, Bristol, UK; n Division of Cancer Epidemiology and Genetics, National Cancer Institute, National Institutes of Health, Bethesda, MD, USA; o Department of Preventive Medicine, Northwestern University Feinberg School of Medicine, Chicago, IL, USA; p Public Health Sciences Division, Fred Hutchinson Cancer Research Center, Seattle, WA, USA; q School of Public Health, University of Washington, Seattle, WA, USA; r Department of Epidemiology, University of Washington School of Public Health, Seattle, WA, USA; s Institute of Environmental Medicine, Karolinska Institutet, Stockholm, Sweden; t University of Southern California, Preventative Medicine, Los Angeles, CA, USA; u Cancer Epidemiology Program, University of Hawaii Cancer Center, Honolulu, HI 96813, USA; v Colorectal Oncogenomic Group, Department of Clinical Pathology, University of Melbourne, Parkville, Victoria, Australia; w Victorian Comprehensive Cancer Centre, University of Melbourne Centre for Cancer Research, Parkville, Victoria, Australia; x Genetic Medicine and Family Clinic, The Royal Melbourne Hospital, Parkville, Victoria, Australia; y Centre for Epidemiology Versus Arthritis, Faculty of Biological Medicine and Health, University of Manchester, Manchester, UK; z Division of Cancer Epidemiology and Genetics, National Cancer Institute, National Institutes of Health, Rockville, MD, USA; aa Institute of Biomedicine, Medical Research Center and Oulu University Hospital, University of Oulu, Oulu, Finland; ab Department of Pediatric Gastroenterology and Metabolic Diseases, Poznan University of Medical Sciences, Poznan, Poland; ac Department of Epidemiology and Biostatistics, MRC Centre for Environment and Health, School of Public Health, Imperial College London, London, UK; ad Unit of Primary Health Care, Oulu University Hospital, OYS, Oulu, Finland; ae Department of Life Sciences, College of Health and Life Sciences, Brunel University London, London, UK; af Institute for Clinical and Translational Research, Baylor College of Medicine, Houston, TX, USA; ag Prosserman Centre for Population Health Research, Lunenfeld-Tanenbaum Research Institute, Sinai Health, Toronto, Canada; ah Dalla Lana School of Public Health, University of Toronto, Toronto, Canada; ai Dementia Research Institute, Imperial College London, London, UK; aj University Hospitals Bristol and Weston NHS Foundation Trust, National Institute for Health Research Bristol Biomedical Research Centre, University of Bristol, Bristol, UK

**Keywords:** Inflammation, Cancer, Mendelian randomization, Genetic epidemiology

## Abstract

**Background:**

Tumour-promoting inflammation is a “hallmark” of cancer and conventional epidemiological studies have reported links between various inflammatory markers and cancer risk. The causal nature of these relationships and, thus, the suitability of these markers as intervention targets for cancer prevention is unclear.

**Methods:**

We meta-analysed 6 genome-wide association studies of circulating inflammatory markers comprising 59,969 participants of European ancestry. We then used combined *cis*-Mendelian randomization and colocalisation analysis to evaluate the causal role of 66 circulating inflammatory markers in risk of 30 adult cancers in 338,294 cancer cases and up to 1,238,345 controls. Genetic instruments for inflammatory markers were constructed using genome-wide significant (*P* < 5.0 × 10^−8^) *cis*-acting SNPs (i.e., in or ±250 kb from the gene encoding the relevant protein) in weak linkage disequilibrium (LD, r^2^ < 0.10). Effect estimates were generated using inverse-variance weighted random-effects models and standard errors were inflated to account for weak LD between variants with reference to the 1000 Genomes Phase 3 CEU panel. A false discovery rate (FDR)-corrected *P*-value (“*q*-value”) <0.05 was used as a threshold to define “strong evidence” to support associations and 0.05 ≤ *q*-value < 0.20 to define “suggestive evidence”. A colocalisation posterior probability (PPH_4_) >70% was employed to indicate support for shared causal variants across inflammatory markers and cancer outcomes. Findings were replicated in the FinnGen study and then pooled using meta-analysis.

**Findings:**

We found strong evidence to support an association of genetically-proxied circulating pro-adrenomedullin concentrations with increased breast cancer risk (OR: 1.19, 95% CI: 1.10–1.29, *q*-value = 0.033, PPH_4_ = 84.3%) and suggestive evidence to support associations of interleukin-23 receptor concentrations with increased pancreatic cancer risk (OR: 1.42, 95% CI: 1.20–1.69, *q*-value = 0.055, PPH_4_ = 73.9%), prothrombin concentrations with decreased basal cell carcinoma risk (OR: 0.66, 95% CI: 0.53–0.81, *q*-value = 0.067, PPH_4_ = 81.8%), and interleukin-1 receptor-like 1 concentrations with decreased triple-negative breast cancer risk (OR: 0.92, 95% CI: 0.88–0.97, *q*-value = 0.15, PPH_4_ = 85.6%). These findings were replicated in pooled analyses with the FinnGen study. Though suggestive evidence was found to support an association of macrophage migration inhibitory factor concentrations with increased bladder cancer risk (OR: 2.46, 95% CI: 1.48–4.10, *q*-value = 0.072, PPH_4_ = 76.1%), this finding was not replicated when pooled with the FinnGen study. For 22 of 30 cancer outcomes examined, there was little evidence (*q*-value ≥0.20) that any of the 66 circulating inflammatory markers examined were associated with cancer risk.

**Interpretation:**

Our comprehensive joint Mendelian randomization and colocalisation analysis of the role of circulating inflammatory markers in cancer risk identified potential roles for 4 circulating inflammatory markers in risk of 4 site-specific cancers. Contrary to reports from some prior conventional epidemiological studies, we found little evidence of association of circulating inflammatory markers with the majority of site-specific cancers evaluated.

**Funding:**

10.13039/501100000289Cancer Research UK (C68933/A28534, C18281/A29019, PPRCPJT∖100005), 10.13039/501100000321World Cancer Research Fund (IIG_FULL_2020_022), 10.13039/501100000272National Institute for Health Research (NIHR202411, BRC-1215-20011), 10.13039/501100000265Medical Research Council (MC_UU_00011/1, MC_UU_00011/3, MC_UU_00011/6, and MC_UU_00011/4), 10.13039/501100002341Academy of Finland Project 326291, European Union's 10.13039/501100007601Horizon 2020 grant agreement no. 848158 (EarlyCause), 10.13039/501100006364French National Cancer Institute (INCa SHSESP20, 2020-076), 10.13039/501100012041Versus Arthritis (21173, 21754, 21755), 10.13039/100000002National Institutes of Health (U19 CA203654), 10.13039/100000054National Cancer Institute (U19CA203654).

**Research in context**

**Evidence before this study**

Preclinical and conventional epidemiological studies have suggested a role of various classes of circulating inflammatory markers in risk of site-specific cancer. These findings suggest that pharmacological targeting of inflammatory markers could be an effective approach for cancer prevention. However, the current evidence base and, thus, the translational utility of these findings to cancer prevention strategies is limited by the unclear relevance of preclinical studies to humans and the susceptibility of conventional observational studies to confounding and reverse causation.

**Added value of this study**

To evaluate the causal relevance of circulating inflammatory markers in risk of adult cancer, we employed a Mendelian randomization design, a quasi-experimental approach which leverages the natural randomization of germline genotype at conception to strengthen causal inference in observational studies. We developed genetic instruments for 66 circulating inflammatory markers by meta-analysing data from 6 genome-wide association studies of these markers in 59,969 participants and tested these for association with risk of 30 adult cancers in 338,294 cancer cases and up to 1,238,345 controls. We found consistent evidence for association of 4 circulating inflammatory markers in risk of 4 site-specific cancers: positive associations between pro-adrenomedullin and breast cancer and interleukin-23 receptor and pancreatic cancer and inverse associations between prothrombin and basal cell carcinoma and interleukin-1 receptor-like 1 and triple-negative breast cancer. Importantly, for 22 of 30 cancer outcomes examined, we found little evidence that any of the circulating inflammatory markers were causally implicated in cancer risk.

**Implications of all the available evidence**

Our comprehensive analyses help to clarify the human biology of inflammatory markers in cancer risk, prioritise further evaluation of select inflammatory markers as potential chemoprevention agents for cancer prevention, and suggest the likely non-causal role of a large and diverse group of inflammatory markers in cancer risk across most anatomical sites examined, deprioritising their further evaluation as targets for cancer prevention.

## Introduction

Emerging evidence implicates chronic inflammation in cancer development.^1^, ^2^, ^3^ Preclinical studies have shown that pro-inflammatory cytokines (e.g., tumour necrosis factor-α, interleukin-1, interleukin-6) promote cancer cell proliferation, invasion, and metastasis, and transcription factors for these markers (e.g., NF-kB and STAT3) are up-regulated across most cancers.^4^, ^5^, ^6^, ^7^, ^8^ Prospective observational studies have reported associations between circulating inflammatory markers and risk of cancer across various anatomical sites.^9^, ^10^, ^11^, ^12^, ^13^, ^14^, ^15^, ^16^, ^17^, ^18^, ^19^, ^20^, ^21^, ^22^, ^23^ Further, pharmacological inhibition of key inflammatory mediators (e.g., COX enzymes, interleukin-1β) in clinical trials has led to reduced risk of site-specific cancers.^24^^,^^25^ These successful trial results suggest that pharmacological targeting of other inflammatory markers identified in the observational epidemiological literature could be an effective approach for cancer prevention.^26^

However, there are important challenges that accompany the translation of findings from observational studies into effective cancer control strategies. This is because of the susceptibility of conventional observational designs to various biases such as residual confounding (e.g., due to unmeasured or imprecisely measured confounders) and reverse causation.^27^^,^^28^ These biases frequently persist despite statistical and methodological efforts to address them,^29^, ^30^, ^31^ making it difficult for observational studies to reliably conclude that a risk factor is causal, and thus a potentially effective intervention target.^32^

Mendelian randomization (MR) uses germline genetic variants as instruments (“proxies”) for risk factors to generate estimates of the effects of these factors on disease outcomes in observational settings.^32^^,^^33^ Since germline genetic variants are quasi-randomly assorted at meiosis and are fixed at conception, MR analyses should be less susceptible to conventional issues of confounding and cannot be influenced by reverse causation bias. In addition, MR analysis considers the long-term effect of risk factors on health outcomes, which is relevant in the context of diseases like cancer where there may be long induction periods between exposure to a particular risk factor and disease initiation.^34^

Previous MR analyses that have examined the association of circulating inflammatory markers with cancer risk have been restricted to examining single inflammatory markers,^35^, ^36^, ^37^, ^38^, ^39^, ^40^, ^41^ individual cancer sites,^36^^,^^37^^,^^42^^,^^43^ or have evaluated the effects of specific classes of inflammatory markers (i.e., cytokines).^44^, ^45^, ^46^ To date, however, no studies have used a systematic approach to comprehensively evaluate different classes of circulating inflammatory markers across adult cancers.

We aimed to systematically evaluate the causal relationship of circulating inflammatory markers with risk of 30 adult cancers. First, we performed a meta-analysis of genome-wide association studies (GWAS) of circulating inflammatory markers to generate novel and stronger genetic instruments for these markers. Second, we used the Open Targets Platform to identify inflammatory markers with prior evidence from preclinical and/or epidemiological studies to support their aetiological role in site-specific cancers and tested relationships of these inflammatory marker-cancer pairs using combined Mendelian randomization and colocalisation analysis (“Validation analyses”). Third, for all remaining inflammatory-marker cancer pairs, we systematically tested their relationship using combined Mendelian randomization and colocalisation analysis to identify potential novel circulating inflammatory markers implicated in cancer risk (“Discovery analyses”).

## Methods

### Identification of inflammatory markers

We compiled a list of all inflammatory markers that corresponded to one or more of the following classes: acute phase proteins, chemokines, growth factors, interferons, interleukins, and tumour necrosis factors.^47^, ^48^, ^49^, ^50^, ^51^, ^52^, ^53^ Inflammatory markers were then mapped to their UniProt ID, resulting in 218 unique markers.^54^

### Identification of GWAS for inclusion in meta-analysis

Between March and May 2021, the GWAS catalog (https://www.ebi.ac.uk/gwas/) and the preprint server bioRxiv (https://www.biorxiv.org/) were searched for genome-wide association studies of circulating proteins for inclusion into the meta-analysis. GWAS of circulating inflammatory markers were included in meta-analyses if they met the following criteria: i) the study was performed in individuals of European ancestry, ii) the study was adjusted for basic covariates only (e.g., age, sex, principal components of genetic ancestry) to avoid issues due to potential collider bias, iii) effect estimates were presented in standard deviation (SD) units or equivalent (e.g., protein concentrations were inverse-normal rank transformed), and iv) complete summary genetic association data were available for *cis*-acting SNPs (i.e., ±250 kb from the gene encoding each marker). We did not contact authors for additional data as our inclusion criteria were to only include studies that posted complete summary genetic association data from the analysis online. Where two studies with >50% sample overlap measured the same protein, we selected the larger study for inclusion into the meta-analysis. In total, 6 studies met all inclusion criteria and 8 were excluded.^55^, ^56^, ^57^, ^58^, ^59^, ^60^ A summary of each included study is presented in Table 1 and a list of excluded studies along with their justification for exclusion is presented in Supplementary Table 1.

**Table 1:** Summary of studies included in GWAS meta-analysis of circulating inflammatory markers.

Study	N proteins	Participants	Units	Adjustment	Protein assay
Karhunen et al.^58^	47 inflammatory proteins	840-13,365 Finnish	Inverse-normal rank transformation	Age, sex, first 10 genetic principal components	Bio-Rad Bio-plex assays, custom panel
Folkersen et al.^55^	90 proteins	30,931 European	NPX values of proteins (on the log2 scale) were rank-based inverse normal transformed and/or standardised to unit variance	Variable across studies	Olink
Gilly et al.^56^	257 proteins	1328 Greek	Inverse-normal transformation of the residuals	Age, age^2^, sex, plate number, per-sample mean NPX value across all assays. Adjustment for season.	Olink
Hillary et al.^57^	70 inflammatory proteins	1936 European older adults	Standardised residuals from these regression models were brought forward for all genetic-protein and epigenetic-protein analyses.	Age, sex, four genetic principal components of ancestry, array plate	Olink
Pietzner et al.^59^	179 proteins	10,708 European	Rank-based inverse normal transformation	Age, sex, sample collection site, 10 principal components	SomaScan
Sun et al.^60^	2995 proteins	3600 European	Rank-inverse normalized	Age, sex, duration between blood draw and processing, first three principal components of ancestry from multi-dimensional scaling	SomaScan

### Data pre-processing and quality control

For each inflammatory marker of interest, UniProt IDs were mapped to proteomic platform-specific IDs based on annotations provided by platform vendors and manual review.^61^ In quality control, 14 markers with the following issues were flagged and removed from one or more studies: those with ambiguous or duplicate Uniprot IDs, unique Uniprot IDs with duplicate probes, genes located on chromosome X, or proteins where summary genetic association data could not be accessed from the relevant data repository (Supplementary Table 2). After removal of problematic inflammatory markers, genetic association data for 204 of 218 markers of interest were available in at least one study. For each of these markers, the relevant protein-coding gene for the marker was identified using the UniProt ID mapping function and genomic coordinates for the gene (build GRCh37) were extracted using BioMart.^62^ Of the 204 inflammatory markers, 116 markers were only measured in one study with the remaining 88 markers taken forward to meta-analysis.

### GWAS meta-analysis to develop genetic instruments for inflammatory markers

Across all studies, summary genetic association data for each marker were extracted for *cis*-acting variants (i.e., ±250 kb from the gene encoding the protein). Genomic coordinates in Gilly et al. were converted from build GRCh38 to GRCh37 prior to data extraction using LiftOver.^63^ All SNPs with a minor allele frequency (MAF) <0.01 and all palindromic SNPs with a MAF >0.40 were removed. For inflammatory markers measured in both Sun et al. and Folkersen et al., summary genetic association data from Sun et al. were not included in meta-analyses due to participant overlap across studies.^55^^,^^60^ Meta-analyses across inflammatory markers were performed using inverse variance-weighted fixed-effects models in METAL.^64^ Of the 88 inflammatory markers included in the meta-analysis, 45 had 1 or more genome-wide significant (*P* < 5.0 × 10^−8^) variants associated with that marker and, therefore, were included in subsequent MR analyses.

### Agreement of SNP effects across studies

To compare agreement of SNP effects across studies included in meta-analyses, standardised effect estimates for inflammatory markers were systematically compared across studies by calculating Pearson correlation coefficients and the percentage of SNPs with effects that were directionally consistent. These analyses were performed by extracting independent *cis*-acting SNPs (r^2^ < 0.01, with reference to the 1000 Genomes Phase 3 CEU Panel) across two *P*-value thresholds (*P* < 5.0 × 10^−8^, *P* < 5.0 × 10^−4^) in PLINK. Pair-wise correlations and directionality comparisons were not performed for Folkersen et al. and Sun et al. due to participant overlap across studies. Comparisons were performed by aligning effect directions of SNPs to the protein-increasing allele to prevent inflated Pearson correlation coefficients.^65^ When performing pair-wise correlations of SNP effects across studies, the median (interquartile range, IQR) agreement in study-level comparison was r = 0.66 (0.24–0.84) when using a *P* < 5.0 × 10^−8^ threshold and r = 0.65 (0.45–0.85) when using a *P* < 5.0 × 10^−4^ threshold. When comparing the percentage of SNPs with effects that were directionally consistent across studies, 93.4% (142/152) were consistent when using a *P* < 5.0 × 10^−8^ threshold and 89.2% (545/611) were consistent when using a *P* < 5.0 × 10^−4^ threshold.

### Genetic instrument construction

Following meta-analysis, genome-wide significant (*P* < 5.0 × 10^−8^) SNPs for each inflammatory marker of interest were extracted and SNPs with evidence of heterogeneity of effect across studies (P_*het*_ < 0.001) were removed. In total, 45 inflammatory markers included in meta-analyses were retained and combined with 21 markers measured in a single study (i.e., not included in the meta-analysis) that had 1 or more *cis*-acting genome-wide significant (*P* < 5.0 × 10^−8^) variant associated with the marker.^66^ Genetic instruments to proxy 66 circulating inflammatory markers were constructed from SNPs that were permitted to be in weak linkage disequilibrium (LD, r^2^ < 0.10), increasing the proportion of variance in each marker explained by the instrument and, thus, maximising instrument strength.^67^

### Cancer GWAS study populations

We obtained summary genetic association data from GWAS of 30 cancer outcomes representing 12 anatomical sites and 18 cancer subtypes within these sites.^68^, ^69^, ^70^, ^71^, ^72^, ^73^, ^74^, ^75^, ^76^, ^77^, ^78^ The median (IQR) number of cases across GWAS of unique anatomical sites was 15,161 (7537–37,344). Analyses in each study were restricted to individuals of European ancestry. Further information on statistical analysis, imputation, and quality control measures for these studies is available in the original publications. A summary of the numbers of cases and controls across each cancer outcome is presented in Supplementary Table 3.

### Analytical approach

We employed a two-stage approach to evaluate the effect of circulating inflammatory markers on cancer risk. We first attempted to validate previously reported inflammatory marker-cancer associations from the preclinical and/or epidemiological literature using the Open Targets platform (“Validation analyses”).^79^ The Open Targets platform integrates data (e.g., gene expression, animal models, text mining, pathways and systems biology) from >20 public sources and uses this data to systematically build an “Overall association score” between drug targets and disease outcomes (i.e., the Open Targets platform generates a summary of the overall evidence implicating a protein-coding gene in a disease outcome). The score is derived by calculating the harmonic sum of the association score by data source weighted by data source weights that aim to calibrate the relevance of each data source relevant to others, independent to their data type categorisation. All inflammatory marker-cancer pairs with an “Overall association score” ≥0.05 were included in validation analyses (scores range from 0 to 1, where 1 represents strong evidence that a protein is implicated in a disease outcome). For the purposes of this analysis, a score of ≥0.05 would suggest, at minimum, some evidence (e.g., across studies evaluating gene expression, animal models, and/or text mining) that a particular inflammatory marker is implicated in a cancer outcome evaluated. Of all remaining inflammatory marker-cancer pairs not included in “Validation analyses”, we then performed a “hypothesis-free” pan-cancer assessment to identify potential novel inflammatory marker-cancer associations (termed “Discovery analyses”).

Mendelian randomization can generate unbiased estimates of causal effects of exposures on disease outcomes if the following assumptions are met: i) the instrument is strongly associated with the exposure (“relevance”), ii) there are no common causes of the instrument and outcome (“exchangeability”), and iii) there is no direct effect of the instrument on the outcome (“exclusion restriction”). Under the assumption of monotonicity (i.e., the direction of the effect of the instrument on the exposure is consistent across all individuals), MR can provide valid point estimates for those participants whose exposure is influenced by the instrument (i.e., complier average causal effect).^80^

For inflammatory markers instrumented by a single SNP, the Wald ratio was used to generate effect estimates and the delta method was used to approximate standard errors. For markers instrumented by two or more SNPs, inverse-variance weighted (IVW) random-effects models were used to estimate causal effects.^81^ Standard errors from IVW models were inflated to account for weak linkage disequilibrium between SNPs by incorporating a correlation matrix using the 1000 Genomes Phase 3 CEU reference panel.^82^^,^^83^

We evaluated the “relevance” assumption by generating estimates of the proportion of variance in each inflammatory marker explained by the instrument (r^2^) and F-statistics. An F-statistic >10 is conventionally used to indicate that instruments are unlikely to suffer from weak instrument bias.^84^ Colocalisation analysis was performed to evaluate whether circulating inflammatory markers and cancer outcomes shared the same causal variant within a locus, necessary but not sufficient to infer causality between these traits. Such an analysis can also permit evaluation of whether circulating inflammatory markers and cancer endpoints are influenced by distinct causal variants that are in linkage disequilibrium with each other, indicative of horizontal pleiotropy (an instrument influencing an outcome through pathways independent to that of the exposure), a violation of the exclusion restriction assumption. Colocalisation analysis was performed by generating ±250 kb windows from the sentinel SNP used to proxy each inflammatory marker. We employed a colocalisation posterior probability (PPH_4_) of >0.70 to indicate support for shared causal variants across circulating inflammatory markers and cancer outcomes. All colocalisation analyses were performed using GCTA-COJO and the coloc package as implemented in Pair-Wise Conditional analysis and Colocalisation analysis (PWCoCo).^85^, ^86^, ^87^ We used default prior probabilities that any SNP within the colocalisation window was associated exclusively with inflammatory marker concentrations (p_1_ = 1 × 10^−4^), exclusively with cancer risk (p_2_ = 1 × 10^−4^), or both traits (p_12_ = 1 × 10^−5^). Finally, iterative leave-one-out analysis was performed iteratively removing one SNP at a time from multi-SNP instruments to examine whether findings were driven by a single influential SNP.

To account for multiple testing, a Benjamini-Hochberg false discovery rate (FDR) correction was applied across “Validation” and “Discovery” analyses separately.^88^ We used an FDR-corrected *P*-value (termed “*q*-value”) threshold of <0.05 to define “strong evidence” to support analyses, with findings between *q*-value ≥0.05 and *q*-value <0.20 defined as “suggestive evidence”.

### Sensitivity analyses to explore potential aptamer or epitope binding effects

Genetic instruments may be associated with circulating protein concentrations due to aptamer or epitope binding artefacts when using protein assays that rely on binding (e.g., SomaScan).^89^ SNP associations in GWAS that employ aptamer-based protein platforms may therefore represent associations with protein measures due to differential binding rather than differences in protein abundance. As variants sensitive to aptamer or epitope binding effects tend to be missense variants, for two studies that used the aptamer-based SomaScan assay (i.e., Sun et al., Pietzner et al.), we flagged all instruments that were missense variants or variants that were in high LD (r^2^ > 0.80) with a missense variant using functional consequence data from the Open Targets platform and the LDlinkR package.^59^^,^^60^^,^^79^^,^^90^ For top findings from “Validation” and “Discovery” analyses generated using multi-SNP instruments, as sensitivity analyses we then re-calculated MR estimates dropping missense variants or variants in high LD with missense variants from instruments. For top findings consisting of single-SNP instruments that were missense variants or in high LD with a missense variant, we explored whether these instruments were also expression quantitative trait loci (eQTL) or splicing quantitative trait loci (sQTL) for the gene encoding the relevant inflammatory marker in the Genotype-Tissue Expression (GTEx) project V8. If missense variants (or variants in high LD with missense variants) also influence expression or alternative splicing of pre-mRNA of the gene encoding the relevant inflammatory marker, causal inference using these variants as instruments is unlikely to be biased even if effect estimates are invalid.^91^

### Replication analysis in the FinnGen study and meta-analysis of results

Replication analyses of findings from “Validation” and “Discovery” analyses were performed using summary genetic association data from the FinnGen study.^92^ FinnGen is a large-scale research project with combined genomic and clinical data on 377,277 participants (release 9). The numbers of cancer cases across replication analyses ranged from 1416 to 15,680 and are presented in Supplementary Table 3. Fixed and random-effects meta-analysis was performed across MR estimates obtained from primary (i.e., “discovery” and “validation” analyses) and replication analyses.

### Expression quantitative trait loci enrichment to examine tissue-specific regulatory mechanisms of effects

For all top findings from “Discovery” and “Validation” analyses we examined whether instruments overlapped with eQTL (*P* < 5.0 × 10^−8^) to identify potential tissue-specific regulatory effects of instruments using data on 15,201 RNA-sequencing samples from 49 tissues of 838 post-mortem donors in the GTEx project V8 and 1544 RNA-sequencing samples from 13 immune cells of 91 healthy subjects in the Database for Immune Cell Expression (DICE).^93^^,^^94^ Where there was eQTL overlap, we then used multiple trait colocalisation (“moloc”) to evaluate colocalisation across circulating inflammatory marker concentrations, tissue-specific or immune cell-specific gene expression, and cancer risk.^95^ The primary objective of these analyses is to identify whether putative effects of circulating inflammatory markers on cancer risk are driven through tissue-specific or immune cell-specific effects of expression of the genes encoding these markers (i.e., facilitating identification of tissue or immune cell-specific regulatory mechanisms that may account for these associations). We employed a colocalisation posterior probability >0.70 to indicate support for shared causal variants across all three traits. We used default prior probabilities that any SNP within the colocalisation window was associated exclusively with inflammatory marker concentrations, tissue-specific or immune cell-specific gene expression, or cancer risk (p_1_ = 1 × 10^−4^); associated with two of these traits (p_2_ = 1 × 10^−6^); or associated with all three traits (p_3_ = 1 × 10^−7^).

### Evaluation of drug repurposing opportunities using DrugBank and clinicaltrials.gov

For all findings showing “strong” or “suggestive evidence” in MR analysis and evidence of colocalisation, we used DrugBank to identify investigational and/or approved drugs targeting these inflammatory markers.^96^ In post-hoc analyses, a clinical trials registry (https://clinicaltrials.gov) was also searched (accessed on 28 November 2023). The availability of drugs targeting these markers could suggest potential for their repurposing as pharmacological agents for cancer prevention.

**Fig. 1:**
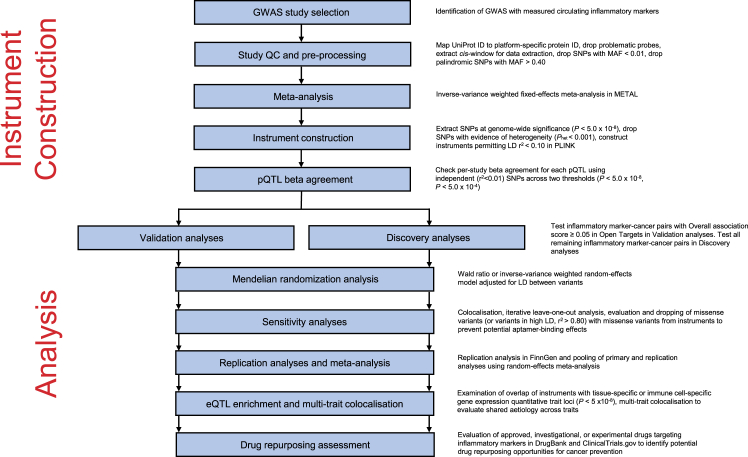
A step-by-step overview of genome-wide association study selection, instrument construction, and statistical analysis stages. GWAS = genome-wide association study, QC = quality control, SNP = single-nucleotide polymorphism, MAF = minor allele frequency, pQTL = protein quantitative trait locus, LD = linkage disequilibrium, eQTL = expression quantitative trait locus.

A step-by-step overview of GWAS selection, instrument construction, and statistical analysis stages is presented in Fig. 1.

### Ethics

This work used summary genetic association data from previously published GWAS. All studies contributing data to these analyses had the relevant institutional review board approval from each country and all participants provided informed consent.

### Role of funders

The funding institutions had no role in the design and conduct of the study; collection, management, analysis, and interpretation of the data; preparation, review, or approval of the manuscript; and decision to submit the manuscript for publication.

## Results

Across 66 circulating inflammatory markers, F-statistics for their instruments ranged from 30.0 to 7608.0, suggesting that instruments were unlikely to suffer from weak instrument bias. Characteristics of genetic variants used to proxy circulating inflammatory markers are presented in Supplementary Table 4. Estimates of r^2^ and F-statistics for each marker are presented in Supplementary Table 5.

### Validation Mendelian randomization analyses

56 of the 66 inflammatory markers were included in validation analyses (i.e., markers with at least one cancer outcome with an overall association score ≥0.05 in Open Targets). In total, 260 target–cancer associations were tested. In Mendelian randomization analysis, there was suggestive evidence to support 6 inflammatory marker-cancer associations. Though categorised as suggestive evidence, the strongest associations observed in this group were for tumour necrosis factor ligand superfamily member 10 (TRAIL) concentrations and breast cancer risk (OR 0.90 per SD increase, 95% CI 0.85–0.95, q-value = 0.072), circulating macrophage migration inhibitory factor (MIF) concentrations and bladder cancer risk (OR 2.46, 95% CI 1.48–4.10, *q*-value = 0.072), and interleukin-7 receptor subunit alpha concentrations and colon cancer risk (OR 0.83, 95% CI 0.74–0.93, *q*-value = 0.093). Findings for all inflammatory marker-cancer associations that employed IVW models were robust to iterative leave-one-out analysis. In colocalisation analysis, there was evidence to support shared causal variants across circulating MIF concentrations and bladder cancer risk in the *MIF* locus (PPH_4_ = 76.1%), but little evidence to suggest shared causal variants across 5 other inflammatory marker-cancer associations. Complete results from primary Mendelian randomization, iterative leave-one-out, and colocalisation analyses are presented in Supplementary Tables 6–8.

### Discovery Mendelian randomization analyses

**Fig. 2:**
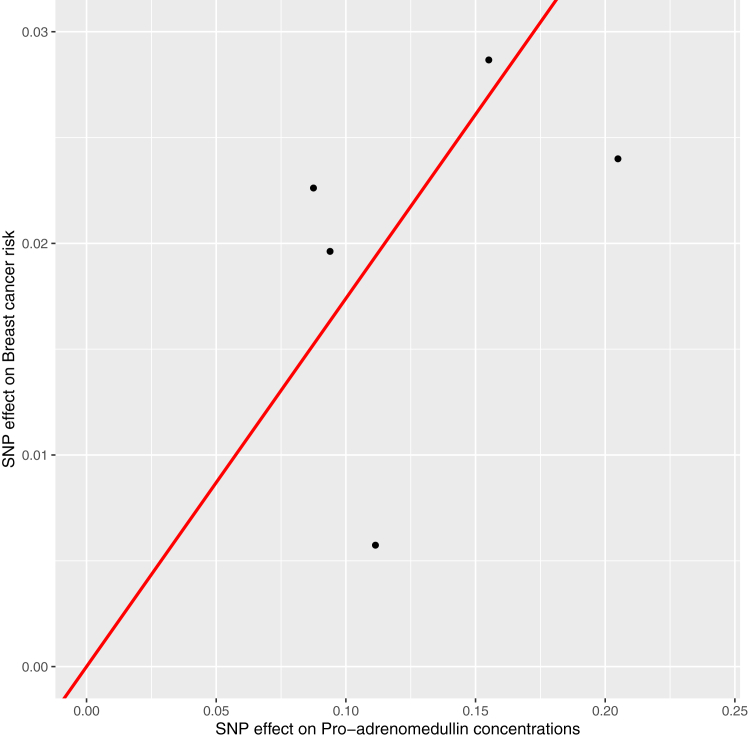
Scatter plot of associations of single-nucleotide polymorphisms with pro-adrenomedullin concentrations and breast cancer risk. SNP = single-nucleotide polymorphism.

**Fig. 3:**
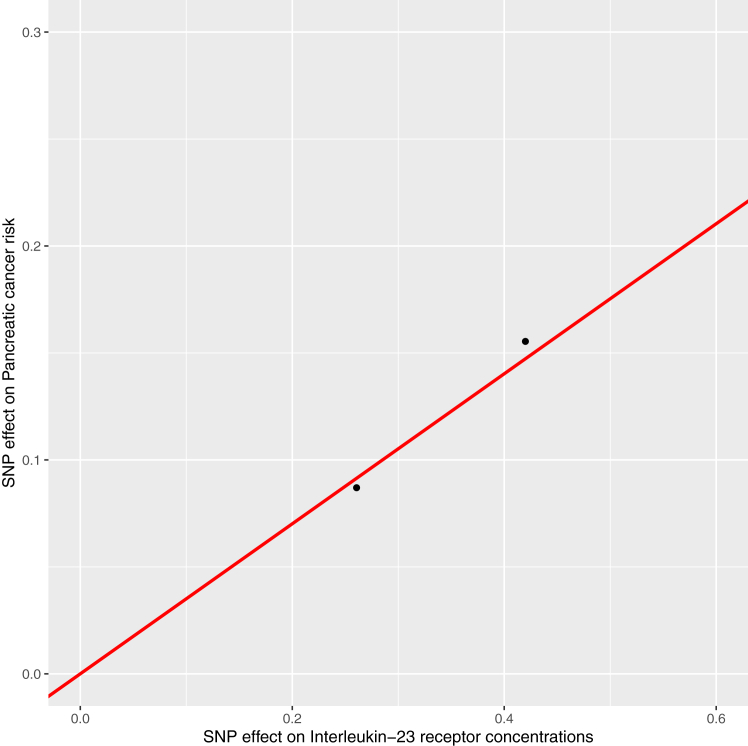
Scatter plot of associations of single-nucleotide polymorphisms with interleukin-23 receptor concentrations and pancreatic cancer risk. SNP = single-nucleotide polymorphism.

**Fig. 4:**
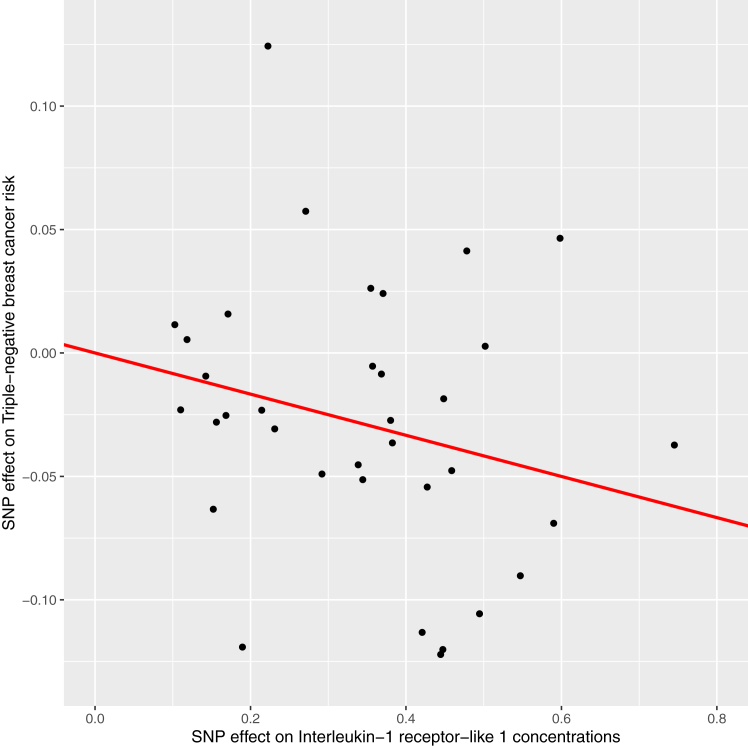
Scatter plot of associations of single-nucleotide polymorphisms with interleukin-1 receptor-like 1 concentrations and triple-negative breast cancer risk. SNP = single-nucleotide polymorphism.

Among the 66 circulating inflammatory markers included in Discovery analyses, there was strong or suggestive evidence for 6 inflammatory marker-cancer associations. The strongest association observed was for circulating pro-adrenomedullin concentrations and breast cancer risk (OR 1.19, 95% CI 1.10–1.29; *q*-value = 0.033). Though categorised as suggestive evidence, the other strongest associations observed in this group were for interleukin-23 receptor concentrations and pancreatic cancer risk (OR 1.42, 95% CI 1.20–1.69; *q*-value = 0.055), prothrombin concentrations and basal cell carcinoma risk (OR 0.66, 95% CI 0.53–0.81; *q*-value = 0.067), serum amyloid P component concentrations and low grade serous ovarian cancer risk (OR 1.86, 95% CI 1.34–2.59; *q*-value = 0.084), and interleukin-1 receptor-like 1 concentrations and triple-negative breast cancer risk (OR 0.92, 95% CI 0.88–0.97, *q*-value = 0.15). Findings for all inflammatory marker-cancer associations that employed IVW models were robust to iterative leave-one-out analysis. In colocalisation analysis, pro-adrenomedullin and breast cancer risk (PPH_4_ = 94.4%), interleukin-23 receptor and pancreatic cancer risk (PPH_4_ = 73.9%), prothrombin and basal cell carcinoma risk (PPH_4_ = 81.8%), and interleukin-1 receptor-like 1 and triple-negative breast cancer risk (PPH_4_ = 85.6%) showed evidence of shared causal variants across traits. Complete findings from primary Mendelian randomization, iterative leave-one-out, and colocalisation analyses are presented in Supplementary Tables 7–9. Scatterplots for analyses of pro-adrenomedullin and breast cancer risk, interleukin-23 receptor and pancreatic cancer risk, and interleukin-1 receptor-like 1 and triple-negative breast cancer risk are presented in Fig. 2, Fig. 3, Fig. 4.

### Replication in FinnGen study and meta-analysis of results

**Fig. 5:**
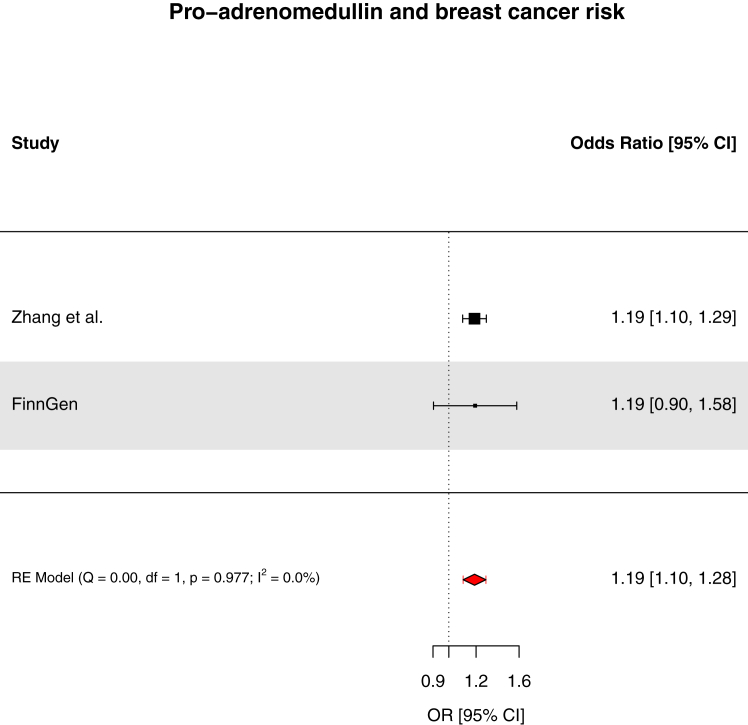
Forest plot of associations of pro-adrenomedullin concentrations and breast cancer risk across Zhang et al. and FinnGen. RE = random effects. Fixed-effects model OR 1.19 (95% CI 1.10–1.28).

**Fig. 6:**
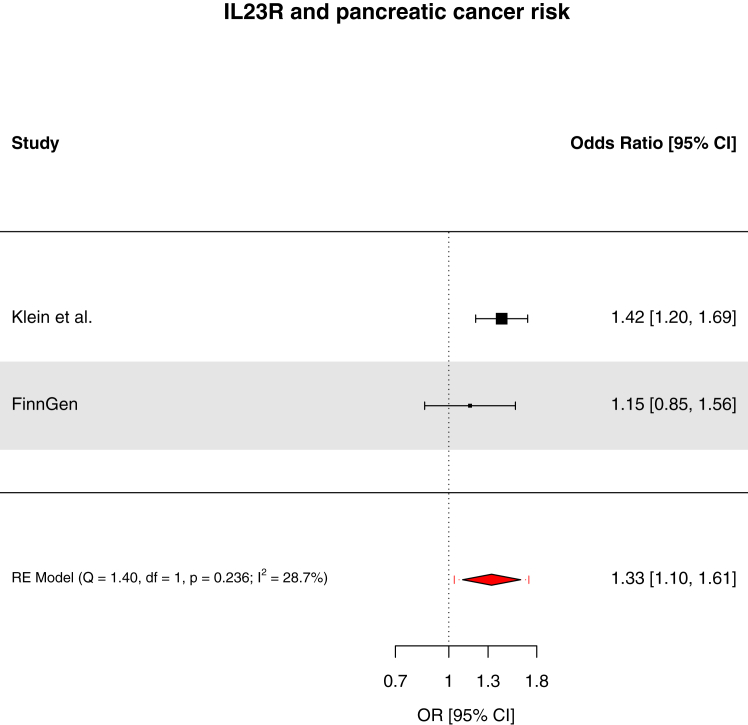
Forest plot of associations of interleukin-23 receptor concentrations and pancreatic risk across Klein et al. and FinnGen. RE = random effects. Fixed-effects model OR 1.35 (95% CI 1.16–1.57).

**Fig. 7:**
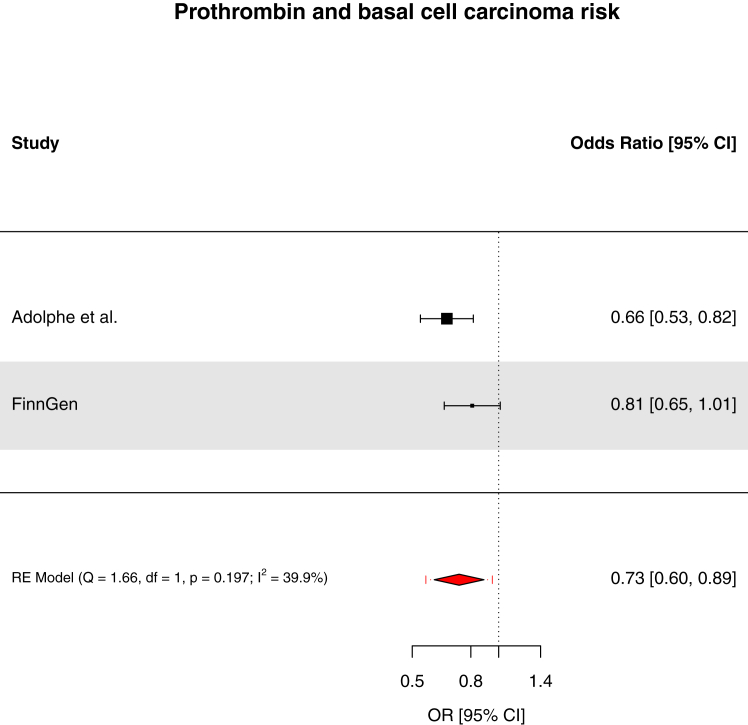
Forest plot of associations of prothrombin concentrations and basal cell carcinoma risk across Adolphe et al. and FinnGen. RE = random effects. Fixed-effects model OR 0.73 (95% CI 0.62–0.85).

**Fig. 8:**
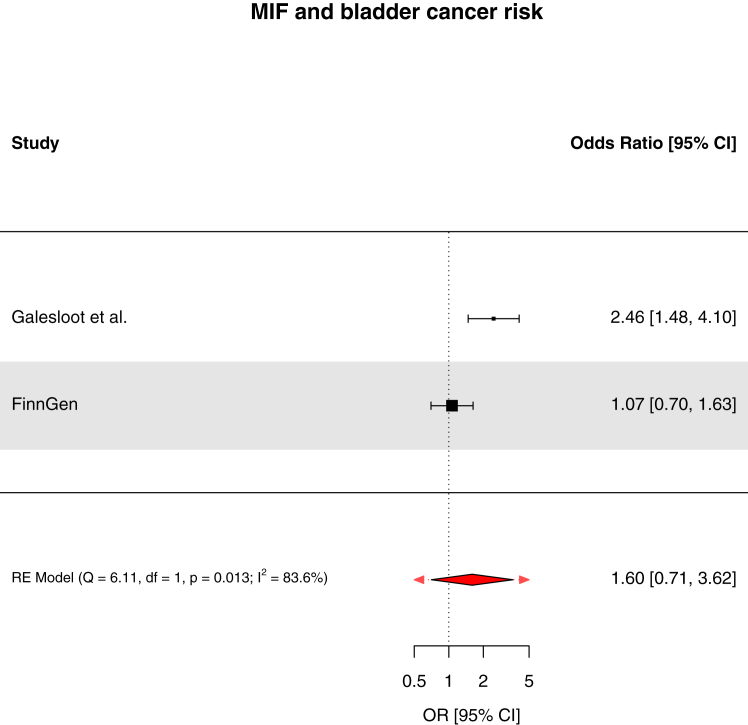
Forest plot of associations of macrophage migration inhibitory factor concentrations and bladder cancer risk across Galesloot et al. and FinnGen. RE = random effects. Fixed-effects model OR 1.50 (95% CI 1.08–2.07).

Replication analyses were performed for all circulating inflammatory marker-cancer associations with strong or suggestive evidence except for interleukin-1 receptor-like 1 and triple-negative breast cancer risk where summary genetic association data could not be identified for this cancer outcome in the FinnGen study or in alternate independent datasets. For 3 of 4 circulating inflammatory markers evaluated in replication analyses, there was consistent evidence of association in pooled analysis with the FinnGen study with either no or low-to-moderate evidence of heterogeneity (I^2^ = 0.0–39.9%) across studies (Fig. 5, Fig. 6, Fig. 7). In replication analysis of the association of macrophage migration inhibitory factor with bladder cancer risk, there was little evidence of association with large heterogeneity (I^2^ = 83.6%) across studies (Fig. 8).

### Evaluation of aptamer or epitope binding effects of instruments

In sensitivity analyses exploring potential aptamer or epitope binding effects of instruments for Mendelian randomization findings showing evidence of colocalisation, one of two SNPs used to instrument interleukin-23 receptor concentrations (rs11581607) was in perfect LD (r^2^ = 1.0) with a missense variant (rs11209026). Mendelian randomization findings were consistent when dropping this SNP from the instrument and calculating a revised causal estimate (OR 1.40, 95% CI 1.10–1.78). In colocalisation analysis employing conditional analysis adjusting for rs11581607, evidence of shared causal variants across interleukin-23 receptor and pancreatic cancer risk associations persisted (PPH_4_ = 69.8%). The SNP used to instrument prothrombin (rs3136516) was in high LD (r^2^ = 0.83) with a missense variant (rs2306029). There was evidence that rs3136516 is a sQTL for *F2* (gene encoding prothrombin) in liver tissue (normalised effect size per copy of circulating prothrombin-increasing allele: 0.63, *P* = 3.1 × 10^−12^). Functional annotation of SNPs used to instrument inflammatory markers along with SNPs in high LD with these variants is presented in Supplementary Table 10.

### Expression quantitative trait loci overlap and multi-trait colocalisation analysis

Across 4 inflammatory markers with consistent evidence of association with cancer outcomes, there was little evidence of eQTL overlap for SNPs used to instrument these markers. Though the association with bladder cancer risk was not consistent in replication analyses in FinnGen, there was evidence that the SNP used to instrument macrophage migration inhibitory factor concentrations (rs2330634) was an eQTL for *MIF* in 35 tissue types. In multiple trait colocalisation analysis, there were low posterior probabilities of shared causal variants across circulating macrophage migration inhibitory factor, tissue-level *MIF* expression, and bladder cancer risk (a summary of eQTL overlap and findings from multiple trait colocalisation analysis is presented in Supplementary Table 11).

### Genetic findings support drug repurposing opportunities in DrugBank and ClinicalTrials.gov

In DrugBank, of the 4 inflammatory markers showing consistent evidence of a causal relationship with site-specific cancer, 3 of these markers (or related markers) are targets of approved or investigational medications. Though there are no approved medications that target the interleukin-23 receptor, three interleukin-23 inhibitors or antagonists (i.e., ustekinumab, tildrakizumab, risankizumab) have been approved to treat autoimmune conditions including moderate-to-severe plaque psoriasis.^97^^,^^98^ Several prothrombin activators or agonists have been approved for the treatment and prevention of bleeding in individuals with haemophilia A (e.g., Moroctocog alfa, Lonoctocog alfa).^99^^,^^100^ Adrecizumab, a monoclonal anti-adrenomedullin antibody, has been evaluated in clinical trials for efficacy in early septic shock (ClinialTrials.gov identifier: NCT03085758), cardiogenic shock (NCT03989531), and coronavirus-19 (NCT05156671). There were no investigational or approved medications targeting interleukin-1 receptor like-1 or the interleukin-1 receptor like-1 ligand interleukin-33 in DrugBank. A summary of investigational and approved medications targeting the 4 inflammatory markers is presented in Supplementary Table 12.

## Discussion

Our systematic Mendelian randomization and colocalisation analysis of 66 circulating inflammatory markers in risk of 30 cancers found consistent evidence for potential roles of 4 markers in risk of 4 site-specific cancers. We identified potential novel associations between four inflammatory markers (pro-adrenomedullin, interleukin-23 receptor, prothrombin, and interleukin-1 receptor-like 1) and risk of site-specific cancers. For 22 of 30 cancer outcomes examined (e.g., overall and subtype-specific lung, ovarian, and endometrial cancer), we found little evidence for association of circulating inflammatory markers with cancer risk despite evidence from conventional epidemiological studies suggesting roles of various markers evaluated in their aetiology.^14^^,^^18^^,^^21^^,^^22^^,^^101^^,^^102^ We also found little evidence that several putative key inflammatory mediators in cancer development (e.g., epidermal growth factor, interleukin-6 receptor, interleukin-8) were associated with cancer risk.^103^, ^104^, ^105^

Some of the disagreement between our findings and those reported in the epidemiological literature could reflect the relatively limited sample size of some cancer outcomes included in our analyses (i.e., 13 of 30 outcomes were restricted to <10,000 cases). Alternatively, conflicting findings could reflect the susceptibility of conventional epidemiological studies to unmeasured or residual confounding (e.g., due to imprecisely measured confounders) and reverse causation (e.g., latent, undiagnosed cancer influencing circulating inflammatory marker concentrations). Assuming that strong and unverifiable assumptions of exchangeability and exclusion restriction hold, our findings therefore do not support widespread effects of those circulating inflammatory markers evaluated on cancer risk across several anatomical sites, though we cannot rule out small to modest or potential time-varying effects of these markers or potential heterogeneity of effects across participant subgroups (e.g., long-term smokers, individuals with autoimmune conditions).

In “Validation analyses” appraising previously reported inflammatory marker-cancer risk relationships, we found suggestive evidence for an association of genetically-proxied macrophage migration inhibitory factor concentrations with bladder cancer risk. MIF is a pleiotropic inflammatory cytokine and critical upstream mediator of innate immunity.^106^^,^^107^ *In vitro* studies of human bladder tissue have supported a role of MIF in tumour cell proliferation.^108^ Further, in animal models of bladder cancer, *MIF* knockout mice displayed decreased angiogenesis and invasion as compared with wild-type mice and those administered oral MIF inhibitors had decreased growth and progression of tumours as compared to controls.^109^^,^^110^ However, our findings did not replicate when pooled with the FinnGen study. This could reflect potential differences in LD structure across participants included in primary and replication analyses (i.e., individuals of Dutch and Finnish ancestry, respectively), or the role of gene-environment interaction or sampling variability across studies. Consequently, the putative role of MIF in development of bladder cancer is unclear and requires further evaluation in future studies.

In “Discovery analyses” examining potential novel inflammatory marker-cancer pairs, we found strong evidence for an association of genetically-proxied pro-adrenomedullin concentrations with breast cancer risk. Pro-adrenomedullin undergoes proteolysis and amidation to yield adrenomedullin and proadrenomedullin N-20 terminal peptide.^111^ Adrenomedullin is a potent vasodilator that is expressed in breast cancer cells, upregulated by hypoxia in these cells, and has been shown to stimulate angiogenesis and tumour proliferation.^112^, ^113^, ^114^ Breast cancer cells overexpressing adrenomedullin show lower levels of various apoptotic factors (e.g., Bax, Bid, caspase 8) and murine models of breast cancer with adrenomedullin overexpression have accelerated bone metastasis and lower rates of survival.^114^^,^^115^ In addition, breast tumour cell-secreted adrenomedullin has been shown to modify cancer-associated adipocytes through paracrine signalling, leading to metabolic changes and lipolysis.^116^

We also found suggestive evidence for an association of genetically-proxied interleukin-23 receptor concentrations with pancreatic cancer risk. The interleukin-23 receptor pairs with the interleukin-12 receptor β-1 subunit to mediate signalling of interleukin-23, a “master regulator” of innate and adaptive immunity and promoter of inflammatory mediators in the tumour microenvironment.^117^^,^^118^ Preclinical studies have reported that interleukin-23 can promote tumour metastasis through up-regulation of angiogenic factors and that interleukin-23 receptor blockade may confer protection against tumour growth.^119^^,^^120^ However, comparison of findings from this Mendelian randomization analysis to these preclinical studies can be complicated by the restriction of the former to evaluation of the effect of interleukin-23 receptor concentrations on pancreatic cancer onset while the latter evaluated the role of interleukin-23 in later stages of tumorigenesis (i.e., tumour growth and metastasis). In addition, serum interleukin-23 concentrations are elevated in patients with pancreatic cancer as compared to controls and higher expression of interleukin-23 in tumour tissues of patients is associated with advanced clinical stage.^121^^,^^122^ Four interleukin-23 inhibitors (i.e., ustekinumab, risankizumab, guselkumab, tildrakizumab) are currently approved for the treatment of immune-mediated inflammatory diseases such as plaque psoriasis.^97^^,^^98^ To date, clinical trials of these medications have not reported links with pancreatic cancer, though sample size (≤1306 participants included in trials) and duration of follow-up of studies (i.e., median 24 weeks to 2.9 years follow-up across studies) have been limited.^123^, ^124^, ^125^, ^126^, ^127^ Though further preclinical and epidemiological work is required to validate and clarify potential mechanisms governing this effect, our findings suggesting an adverse association of genetically-proxied interleukin-23 receptor concentrations with pancreatic cancer risk provide tentative support for a potential role for interleukin-23 inhibition or antagonism as a pharmacological approach for pancreatic cancer prevention.

Finally, there was suggestive evidence to support protective associations of genetically-proxied prothrombin concentrations with basal cell carcinoma and interleukin-1 receptor-like 1 concentrations with triple-negative breast cancer risk. Prothrombin is proteolytically cleaved to form thrombin, the end-product of the coagulation cascade that converts soluble fibrinogen to a fibrin clot. Preclinical studies have reported that thrombin can induce tumour growth, metastasis, and angiogenesis and that thrombin inhibitors suppress tumour growth and metastasis in some cancer cell lines.^128^, ^129^, ^130^ Our analyses evaluated the effect of circulating prothrombin in onset of basal cell carcinoma and, therefore, are not necessarily inconsistent with a possible role of prothrombin in driving later stages of tumorigenesis (e.g., akin to the hypothesised opposing roles of folate in cancer development and progression).^131^^,^^132^ In our analyses, the SNP used to instrument prothrombin was in high LD with a missense variant which could influence aptamer binding and produce biased effect estimates. Though the SNP used to instrument prothrombin was also a sQTL for *F2* (gene encoding prothrombin) in liver tissue, confident causal conclusions about the direction and magnitude of an effect of genetically-proxied prothrombin concentrations on cancer risk mediated via this variant cannot be made. Interleukin-1 receptor-like 1 is the cognate receptor for interleukin-33, an epithelial-derived cytokine which has been reported to confer both pro- and anti-tumorigenic effects, depending on the tumour and cellular context, expression levels, and the nature of the inflammatory environment.^133^^,^^134^ Few studies have examined a potential role of interleukin-33 in triple-negative breast cancer risk and findings from these studies have variably found that interleukin-33 expression is higher in triple-negative breast cancer cell lines as compared to luminal cell lines and that increased expression of this marker is associated with improved survival in triple-negative breast cancer risk.^135^^,^^136^ The potential protective role of circulating interleukin-33 concentrations in triple-negative breast cancer risk requires further examination in future studies.

Strengths of this analysis include the comprehensive and systematic evaluation of a large number of circulating inflammatory markers, representative of different classes of inflammatory markers, in risk of 30 adult cancers. We used a dual “hypothesis-validating” and “hypothesis-generating” approach to attempt to validate previously reported inflammatory marker-cancer pairs and to identify potential novel inflammatory marker-cancer pairs. By performing a meta-analysis of 6 prior GWAS of circulating inflammatory marker concentrations we were able to generate stronger *ci*s-acting instruments for inflammatory markers examined and to develop novel instruments for some markers, increasing statistical power and the breadth of analyses performed. The use of colocalisation as a sensitivity analysis permitted us to test the robustness of Mendelian randomization findings to confounding through linkage disequilibrium. Only 5 of 12 Mendelian randomization findings with “strong” or “suggestive” evidence of association were consistent in colocalisation analyses, highlighting the importance of evaluation of shared causal variants as further support for causality of traits examined, though we cannot rule out low power influencing findings from some of these analyses.

There are several limitations to these analyses. First, the presence or absence of associations of circulating inflammatory markers may not reflect potential tissue-specific effects of these markers in cancer development. While we examined tissue-specific regulatory mechanisms underpinning effects of inflammatory markers showing strong or suggestive evidence of association with cancer risk, we did not systematically evaluate potential tissue-specific effects of genes encoding inflammatory markers across all cancer endpoints. Second, Mendelian randomization estimates represent the effect of long-term genetically-proxied inflammatory marker concentrations on cancer risk which may not correspond to the effect of pharmacological inhibition of these markers over relatively limited periods of time in clinical trials. Third, effect estimates presented assume no gene-environment or gene–gene interactions and linear and time-fixed effects of markers on cancer risk. Fourth, our analyses were restricted to cancer risk and not progression and therefore may not be informative of the utility of targeting inflammatory markers examined in the context of cancer treatment. Fifth, our analyses were performed in individuals of European ancestry and therefore the generalisability of these findings to non-European populations is unclear. Sixth, statistical power was likely limited for some rarer cancers and select cancer subtypes. Seventh, though various sensitivity analyses were employed to test robustness of our findings to potential violations of exchangeability and exclusion restriction assumptions, these assumptions are unverifiable. Eighth, we cannot rule out the possibility that the association of genetically-proxied prothrombin concentrations with basal cell carcinoma risk is driven through aptamer-binding effects given that the variant used to instrument this marker is in high LD with a missense variant. Ninth, though F-statistics calculated across instruments suggest that weak instrument bias is unlikely, the use of LD pruning for instrument construction can lead to less reliable inferences in the presence of weak instruments as compared to, e.g., factor analysis or Bayesian variable selection approaches.^137^ Finally, the inflammatory markers included in this analysis constitute a non-exhaustive list of inflammation-related markers which may influence cancer risk.

We found limited overlap of instruments for circulating inflammatory markers with tissue-specific or immune cell-specific eQTLs for the genes encoding these proteins which could plausibly reflect genetic effects on processes other than transcription, including protein degradation, binding, secretion, or clearance from circulation.^60^ Systematic evaluation of the role of tissue-specific eQTLs for inflammation-related genes in cancer risk could provide further insight into tissue-level regulatory mechanisms influencing cancer development. There is a need to further replicate and validate findings, particularly for novel associations linking inflammatory markers to cancer risk identified in “Discovery” analyses. Future studies restricted to participant sub-groups with elevated risk of cancer (e.g., life-long smokers, individuals with chronic inflammatory conditions) could aid in identification of inflammatory markers mediating cancer risk in these groups. Finally, evaluation of the potential role of circulating inflammatory markers in cancer prognosis could inform on the possible utility of the pharmacological targeting of these markers as cancer treatment.

## Contributors

All authors read and approved the final version of the manuscript. JY and KKT have verified the underlying data.

Conceptualisation: JY; Formal Analysis: JY, JWR; Data curation: JY, DM, VH, ND, NM, KB, TEG, LAK, SV, M-RJ, CIA, RJH, AD, MJ, MJG, KKT; Writing - original draft: JY; Writing - review & editing: JY, JWR, DM, VK, JH, ND, NM, KB, EB, KS-B, SJL, TEG, LAK, SV, PM, DA, LH, PAN, EW, AW, AHL, LLM, AIP, DDB, the International Lung Cancer Consortium, the PRACTICAL consortium, SSZ, DG, SJC, MPP, GDS, PB, K-HH, M-RJ, CIA, RJH, AD, MJ, MJG, KKT, RMM.

## Data sharing statement

Complete summary genetic association data from GWAS meta-analyses of 66 circulating inflammatory markers will be made available on the GWAS catalog ((https://www.ebi.ac.uk/gwas/) upon publication of this manuscript.

We obtained summary genetic association data on breast cancer risk from the Breast Cancer Association Consortium (https://bcac.ccge.medschl.cam.ac.uk/), ovarian cancer risk from the Ovarian Cancer Association Consortium (https://ocac.ccge.medschl.cam.ac.uk/), endometrial cancer risk from the Endometrial Cancer Association Consortium (https://www.ebi.ac.uk/gwas/publications/30093612#study_panel), non-Hodgkin lymphoma risk from Burrows et al. (10.5523/bris.aed0u12w0ede20olb0m77p4b9), and basal cell carcinoma risk from Adolphe et al. (https://www.ebi.ac.uk/gwas/publications/33549134#study_panel). Approval was received to use restricted summary genetic association data from GECCO, INTEGRAL ILCCO, and PRACTICAL consortia after submitting a proposal to access this data. Summary genetic association data from these consortia can be accessed by contacting GECCO (kafdem@fredhutch.org, INTEGRAL ILCCO (rayjean.hung@lunenfeld.ca) (https://ilcco.iarc.fr/), and PRACTICAL (practical@icr.ac.uk). Approval was also received to use restricted summary genetic association data on pancreatic cancer risk via dbGaP release phs000206.v5.p3. To enquire about gaining access to summary genetic association data for renal and head and neck cancer risk, contact brennanp@iarc.fr. To enquire about gaining access to summary genetic association data for bladder cancer risk, contact bart.kiemeney@radboudumc.nl.

## Declaration of interests

GDS reports Scientific Advisory Board Membership for Relation Therapeutics and Insitro. Where authors are identified as personnel of the International Agency for Research on Cancer/World Health Organization, the authors alone are responsible for the views expressed in this article and they do not necessarily represent the decisions, policy or views of the International Agency for Research on Cancer/World Health Organization.

## Appendix A Supplementary data

Supplementary_Tables

Supplementary_Note
